# Unveiling the role of UGT enzymes in chemoresistance: a path to enhanced cancer pharmacotherapy

**DOI:** 10.20517/cdr.2026.10

**Published:** 2026-06-08

**Authors:** Gesine Kretzschmar, Youssif Budagaga, Lisa Groß, Jan-Heiner Küpper, Sarah Kammerer, Jakub Hofman

**Affiliations:** ^1^Institute of Biotechnology, Brandenburg University of Technology Cottbus-Senftenberg, Senftenberg 01968, Germany.; ^2^Department of Pharmacology and Toxicology, Faculty of Pharmacy in Hradec Králové, Charles University, Hradec Králové 500 05, Czech Republic.; ^#^These authors share senior authorship.

**Keywords:** UDP-glucuronosyltransferase, drug resistance, cancer, chemotherapy, tyrosine kinase inhibitor

## Abstract

**Aim:** Chemotherapeutic resistance is a leading cause of cancer-related mortality worldwide. It has been suggested to be at least partly driven by the activity of uridine diphosphate glucuronosyltransferases (UGTs). However, this hypothesis is primarily supported by indirect evidence with potential confounding factors arising from the use of multienzymatic models among other things. Here we present new HepG2 models transduced with UGT1A1 or UGT2B7 that provide mechanistic insights into the potential role of UGTs in resistance to SN-38, etoposide, and epirubicin.

**Methods:** The new cellular models were characterised by immunofluorescence, quantitative real-time PCR, and doubling-time assessment. Possible differences in chemotherapeutic efficacy were evaluated using comparative ATP viability assays and an inhibitor-based verification study. Drug combination assays were conducted to determine whether dual-activity modulation could combat enzyme-mediated resistance, using the quantitative Chou-Talalay method to detect synergistic effects. Finally, an advanced physiologically relevant model based on proliferating primary upcyte hepatocytes was characterised and used to verify the results obtained with the transduced models.

**Results:** The comparative ATP viability assays and verification studies using the UGT inhibitors atazanavir and diclofenac confirmed that both UGT enzymes significantly reduced the tested drugs’ pharmacodynamic activity. Drug combination experiments then showed that UGT1A1 activity can be synergistically targeted by nilotinib and regorafenib via a new dual-activity modulation approach. Finally, the role of UGT1A1 in resistance to SN-38 was confirmed in a complex model based on primary upcyte hepatocytes.

**Conclusion:** After *in vivo* confirmation, our findings could potentially be translated into effective and safe combination regimens for oncology patients.

## INTRODUCTION

Phase II biotransformation enzymes are key metabolic transferases that are mainly expressed in the human liver and intestine. They conjugate drugs, xenobiotics, and their phase I metabolites with endogenous compounds that increase their , thereby increasing hydrophilicity, facilitating their excretion from the body^[1]^. The uridine diphosphate glucuronosyltransferases (UGTs) are a major superfamily responsible for phase II drug metabolism. As two of the most clinically relevant isoforms within this superfamily, UGT1A1 and UGT2B7 are important sites for pharmacokinetic drug-drug interactions^[2]^. UGTs are bound to the membrane of the endoplasmic reticulum and catalyze the transfer of glucuronic acid to nucleophilic functional groups (-OH, ‐SH, ‐NH_2_, ‐COOH, or activated carbon centers) on their substrates. Glucuronidated metabolites typically exhibit significantly lower pharmacological activity than their parent compounds because glucuronidation represents a significant structural change^[2]^. It has therefore been hypothesized that phase II enzyme-mediated metabolism may contribute to pharmacokinetic anticancer drug resistance^[3]^. Such resistance may arise by two mechanisms. Systemic resistance resulting from reduced bioavailability and accelerated drug elimination is most likely to occur in individuals with high liver/intestine UGT expression or those experiencing UGT induction due to drug-drug interactions, whereas tumour-specific resistance may arise from direct enzymatic deactivation of drugs within cancer cells, particularly those with detectable UGT expression. Although UGT levels vary across tumour types and individuals, some isozymes exhibit significant upregulation in specific cases^[3,4]^. However, there is currently no direct experimental evidence of intratumoural UGT-mediated drug resistance. Conclusions on these issues have therefore been based largely on indirect evidence or are complicated by confounding factors such as the use of multienzymatic models^[3]^.

Only a limited subset of chemotherapeutics inactivated by metabolism can be considered prone to intratumoural enzyme-mediated resistance. Drugs within this subset are described as sensitive resistance victims, and they have two defining characteristics: (1) a high intrinsic clearance rate; and (2) deactivation by enzymes that are also key actors in their overall metabolic degradation. The second criterion is particularly important because it affects the feasibility of modulating this type of pharmacokinetic resistance. A detailed literature review revealed that the chemotherapeutic topoisomerase inhibitors epirubicin, etoposide, and SN-38 may satisfy both criteria^[5-7]^. All three are efficiently inactivated via UGT enzymes: SN-38, the active form of irinotecan, is metabolized by UGT1A1, epirubicin by UGT2B7, and etoposide by both isoforms^[5-8]^.

Here we present the development and characterisation of a HepG2 model with stable protein-level overexpression of UGT1A1 or UGT2B7. This “single gene”-transduced cellular model was used to investigate the roles of these enzymes in resistance to epirubicin, etoposide, and SN-38. Comparative cell viability assays using UGT-transduced and empty vector (EV)-transduced control cells were performed initially, and the observed resistance patterns were verified in assays using the known UGT inhibitors atazanavir and diclofenac. The potential of UGT1A1 as a target for dual-activity resistance modulation was then examined in resistance-combatting assays using the tyrosine kinase inhibitors (TKIs) nilotinib and regorafenib. Finally, we compared the results obtained with the HepG2 model to those obtained in experiments using proliferating primary-like human upcyte hepatocytes.

## METHODS

### Reagents and chemicals

The cytostatic SN-38 was obtained from Selleckchem, USA. Etoposide and epirubicin hydrochloride came from MedChemExpress, USA. The UGT2B7-preferential inhibitor diclofenac (diethylamine) was obtained from Cayman Chemical, USA. Ketoconazole, thiazolyl blue tetrazolium bromide, dimethyl sulfoxide (DMSO) for molecular biology, fetal bovine serum (FBS), the UGT1A1 inhibitor atazanavir, phosphate buffered saline (PBS), Triton X-100, and bovine serum albumin (BSA) were sourced from Merck, USA. Blasticidin was purchased from PAA, GE Healthcare, Austria and the lentiviral expression vector from Life Technologies GmbH, Germany. Opti-MEM was bought from Gibco, USA. The cDNA Synthesis Revert Aid H Minus Reverse Transcriptase Kit, the Maxima Probe qPCR Master Mix, and the BCA Protein Assay Kit were purchased from Thermo Fisher Scientific, USA. The CellTiter-Glo® 2.0 Cell Viability Assay Kit and the UGT Activity Assay/Ligand Screening Kit were obtained from Promega, USA and Abcam, UK, respectively. The DNase Digestion Kit was from Invitrogen, USA, and the Innu-Prep Mini Kit was from Analytic Jena, Germany. Primers for quantitative real-time polymerase chain reaction (qRT-PCR) were obtained from BioTez, Germany. Antibodies for immunofluorescence were supplied by Santa Cruz Biotechnology, USA. The anti-UGT2B7 antibody was obtained from Cusabio Biotech, USA. Dulbecco’s Modified Eagle’s Medium (DMEM) and L-Glutamine Stable 100X 200mM were from Biowest, France. Upcyte® hepatocyte culture medium and upcyte® hepatocyte high performance medium were purchased from upcyte® technologies, Germany. Rat tail collagen type I was bought from Corning, USA. The innuPREP RNA Mini Kit 2.0 was purchased from Innuscreen, USA. EvaGreen was obtained from Biotium, USA. 4′,6-diamidino-2-phenylindole (DAPI) and agarose were from Carl Roth, Germany.

### Generation of UGT1A1- and UGT2B7-overexpressing HepG2 cells

A “single gene”-transduced HepG2 model was generated and characterized for use in studying UGT-mediated resistance. To generate UGT1A1- and UGT2B7-overexpressing HepG2 clones, human *UGT1A1* or *UGT2B7* complementary DNA (cDNA; NCBI reference sequences: NM_000463 and NM_001074) was subcloned into a lentiviral expression vector as described previously^[9]^. Recombinant lentiviruses were then generated according to the manufacturer’s instructions and used to infect parental HepG2 cells (American Type Culture Collection, USA). EV-transduced cell clones without cDNA in the expression vector were also produced using the same procedure to serve as controls. Infected cells were selected with 3 µg/mL blasticidin. Surviving clones were isolated to establish a single-cell derived population, propagated, and screened for *UGT*/UGT overexpression by qRT-PCR/immunofluorescence as described below. Population doubling times (PDT) were determined by seeding cells into 6-well plates at a density of 6 × 10^5^ cells/well and culturing them under standard conditions. A half medium change was performed after 4 days and then repeated every second day thereafter. Cells were harvested by trypsinization and counted daily for 10 days using a Neubauer counting chamber. PDT were determined using the equation PDT = (t2 - t1) × log2/log(n2/n1), where t2 - t1 is the time interval of the linear growth rate in h, n1 is the cell count at t1, and n2 is the cell count at t2.

### Cell culture

The overexpressing cell lines HepG2-EV (empty vector transduced), HepG2-UGT1A1, and HepG2-UGT2B7 were cultivated in DMEM supplemented with 10% FBS and L-glutamine at 37 °C and 5% CO_2_ in a humidified incubator. The cells were passaged every 3-4 days at 70% confluence. Morphological images were acquired with a CKX41 microscope (Olympus, Germany).

Primary-like proliferation-competent human hepatocytes 653-03 were supplied by upcyte® technologies, Germany, and the HC10-45 B3 and HepaFH3 upcyte cell lines were generated by lentiviral transduction as described by Herzog *et al.* and Burkard *et al.*^[10,11]^. Cells were cultivated in hepatocyte culture medium at 37 °C and 5% CO_2_ in a humidified incubator on collagen coated surfaces (50 µg/mL collagen type I in 0.08 N acetic acid). In preparation for experiments, the medium was replaced with hepatocyte high performance medium 2-3 days before the start of the experiment. The cells were passaged every 5-7 days at a confluence of 90%. Mycoplasma infection was routinely tested for in all cell cultures. Cells with passage counts of 10-25 were used in all experiments.

### ATP cell viability assay and drug combinations

HepG2 cells were seeded at 1 × 10^4^ cells/well and upcyte hepatocytes at 4.5 × 10^4^ cells/well on 96-well culture plates. After cultivation for 24 h, the cells were treated with different concentrations of a drug or drug combinations in the appropriate culture media for 24, 48, or 72 h (for comparative experiments with single drugs) or 72 h (for drug combinations). A vehicle DMSO control and 60% DMSO served as 100% and 0% viability controls, respectively. After the chosen incubation time, ATP assays were performed with a CellTiter-Glo® 2.0 Cell Viability Assay kit according to the manufacturer’s instructions. Luminescence readings were performed using an Infinite M200 Pro microplate reader (Tecan, Switzerland) with an integration time of 250 ms. The background luminescence, recorded from a set of cell-free wells, was deducted from the luminescence signals of the drug-treated wells. For resistance reversal assays with TKIs, the viability data were transformed into fraction of cells affected (F_A_) values. F_A_ values represent the percentage of cells killed under the applied conditions and were calculated as 100 - remaining cell viability (%). The F_A_ values and the corresponding drug concentrations were then used to compute the Chou-Talalay combination index (CI), enabling accurate quantification of drug combination effects. CI values were calculated using the CompuSyn 3.0.1 software package (ComboSyn Inc., Paramus, NJ, USA) and used to classify the effects of the combined drug treatments as synergistic (CI < 0.9), additive (CI = 0.9-1.1), or antagonistic (CI > 1.1)^[12]^.

### qRT-PCR

RNA was isolated from cell pellets using the innuPREP RNA Mini Kit 2.0 according to the manufacturer’s instructions. DNase digestion was performed with the DNase Digestion Kit according to the manufacturer’s instructions. RNA integrity was confirmed via 1% agarose gel electrophoresis. cDNA synthesis was performed using the cDNA Synthesis Revert Aid H Minus Reverse Transcriptase Kit according to the manufacturer’s instructions. Reverse transcription was conducted using the RevertAid reverse transcriptase and 1 µg of DNase-digested RNA. *ABCG2* (forward sequence: 5’-TGGCTTAGACTCAAGCACAGC-3’; reverse: 5’-TCGTCCCTGCTTAGACATCC-3’), *ABCC2* (forward sequence: 5’-GTCAGAAGCAGCGGATCAGC-3’; reverse: 5’-TCCTCTTCAGGGCCTGTATG-3’), *ABCB1* (forward sequence: 5’-ACAGAAAGCGAAGCAGTGGT-3’; reverse: 5’-ATGGTGGTCCGACCTTTTC-3’), *UGT1A1* (forward sequence: 5’-CTCTCCTCTCATTCAGATCAC-3’; reverse: 5’-CAAAGTCACTTCTAAACAGCC-3’) and *UGT2B7* (forward sequence: 5’-GGTGTTTTCTCTGGGGTCAA-3’; reverse: 5’-TCCCATCAAATCTCCACAGA-3’) mRNA levels were determined using Maxima Probe qPCR Master Mix and EvaGreen in 96-well plates according to the manufacturer’s instructions, amplifying 0.3 µL of cDNA per reaction in 10 µL reaction volumes. qRT-PCR was performed using a CFX96 Touch Real-Time PCR Detection System (Bio-Rad, Hercules, CA, USA) with initial denaturation at 95 °C for 3 min and 40 repeats of a cycle of 95 °C for 10 s, 59 °C for 10 s, and 72 °C for 30 s, followed by melting curve analysis. Relative quantification of the examined genes was performed using the 2^-ΔΔCt^ method. *GAPDH* (forward sequence: 5’-TGCACCACCAACTGCTTAGC-3’; reverse: 5’-GGCATGGACTGTGGTCATGAG-3’) and *SDHA* (forward sequence: 5’-CGAACGTCTTCAGGTGCTTT-3’; reverse 5’-AAGAACATCGGAACTGCGAC-3’) levels were used as an internal control to normalize the variability in expression levels.

### Immunofluorescence

Cells were seeded on 96-well plates at 4.5 × 10^4^ cells/well and incubated for 24 h. The medium was then replaced with ice-cold methanol for fixation, after which the plate was incubated for 5 min at room temperature followed by two washing steps with PBS. Cells were blocked with 10% BSA in PBS for 30 min at room temperature followed by three washing steps with PBS, after which a primary antibody in 1.5% BSA in PBS was applied; the mouse anti-UGT1A primary antibody was used at 1:200 while the rabbit anti-UGT2B7 antibody was used at 1:50. The cells were then incubated at 4 °C overnight. The antibody solution was removed on the following day and the cells were washed 3 times for 5 min with PBS. A Cy3-anti-mouse or anti-rabbit secondary antibody was then applied at 1:200 dilution with DAPI at 1:10,000 dilution in 1.5% BSA in PBS. After incubation at room temperature for 60 min in darkness, the cells were washed again 3 times for 5 min each with PBS. The cells were then covered with PBS and imaged with a BZ-X800 fluorescence microscope using the associated BZ-X800 software package supplied by Keyence (Osaka, Japan). The same exposure settings were used for each antibody in all experiments. UGT1A1/UGT2B7 expression was quantified by analysing the mean red fluorescence intensity (MFI)/cell using the QuPath software^[13]^.

### UGT activity assessment

HepG2-EV, HepG2-UGT1A1, and HepG2-UGT2B7 cells were cultivated under standard conditions as described above and used to prepare enzyme-containing microsomal fractions. Cells were lysed in buffer containing 25 mM Tris-HCl, 150 mM NaCl, 0.25% Triton X-100, and 10% glycerol (pH = 7.6). Cell debris and nuclei were removed by centrifugation at 12,000 × *g* for 10 min at 4 °C, and the resulting supernatant was ultracentrifuged at 105,000 × *g* for 60 min at 4 °C to isolate the microsomal fraction. The microsomal pellet was resuspended in a 0.1 M potassium phosphate buffer with 50 mM potassium chloride, 1.1 mM ethylenediaminetetraacetic acid, 0.1 mM dithiothreitol, 0.5 mM phenylmethylsulfonyl fluoride, and 20% (v/v) glycerol (pH 7.5). The protein concentration was determined using a BCA Protein Assay Kit and UGT activity was assessed using a fluorescence-based UGT Activity Assay/Ligand Screening Kit according to the manufacturer’s instructions. Assays were performed in black 96-well plates at a final volume of 100 µL/well. The reactions for the activity assay were performed using 50 µL of 2 × sample premix containing a microsomal protein sample (0.05 mg/well), 10 µL of 10 × substrate working solution, and 20 µL assay buffer, with an extra 20 µL of assay buffer for controls without uridine diphosphate glucuronic acid (UDPGA). Alamethicin was added to the samples, after which they were incubated on ice for 15 min. The plates were then incubated at 37 °C for 5 min and reactions were initiated by adding 20 µL of 5 × UDPGA. Fluorescence was measured immediately at excitation/emission wavelengths of 415/502 nm in kinetic mode for 40 min. UGT activity was calculated from the linear phase of the reaction progress curves after subtracting the UDPGA-free blank and is reported in units of specific activity (pmol substrate glucuronidated/min/mg protein).

### Statistical analysis

Statistical analyses were performed using GraphPad Prism 8 (GraphPad Software Inc., USA). IC_50_ values were calculated by nonlinear regression. Differences between two groups were compared with Student’s *t*-test. Comparisons involving more than two groups were done by one-way analysis of variance (ANOVA) with Tukey’s post-test for multiple comparisons. The following symbols denote specific significance levels: ns = not significant (*P* > 0.05), ^*^P < 0.05, ^**^P < 0.01, ^***^P < 0.001, and ^****^ *P* < 0.0001. At least three independent replicates were conducted per experiment, each with biological triplicates.

## RESULTS

### Characterisation of UGT1A1- and UGT2B7-overexpressing HepG2 cells

**Figure 1:**
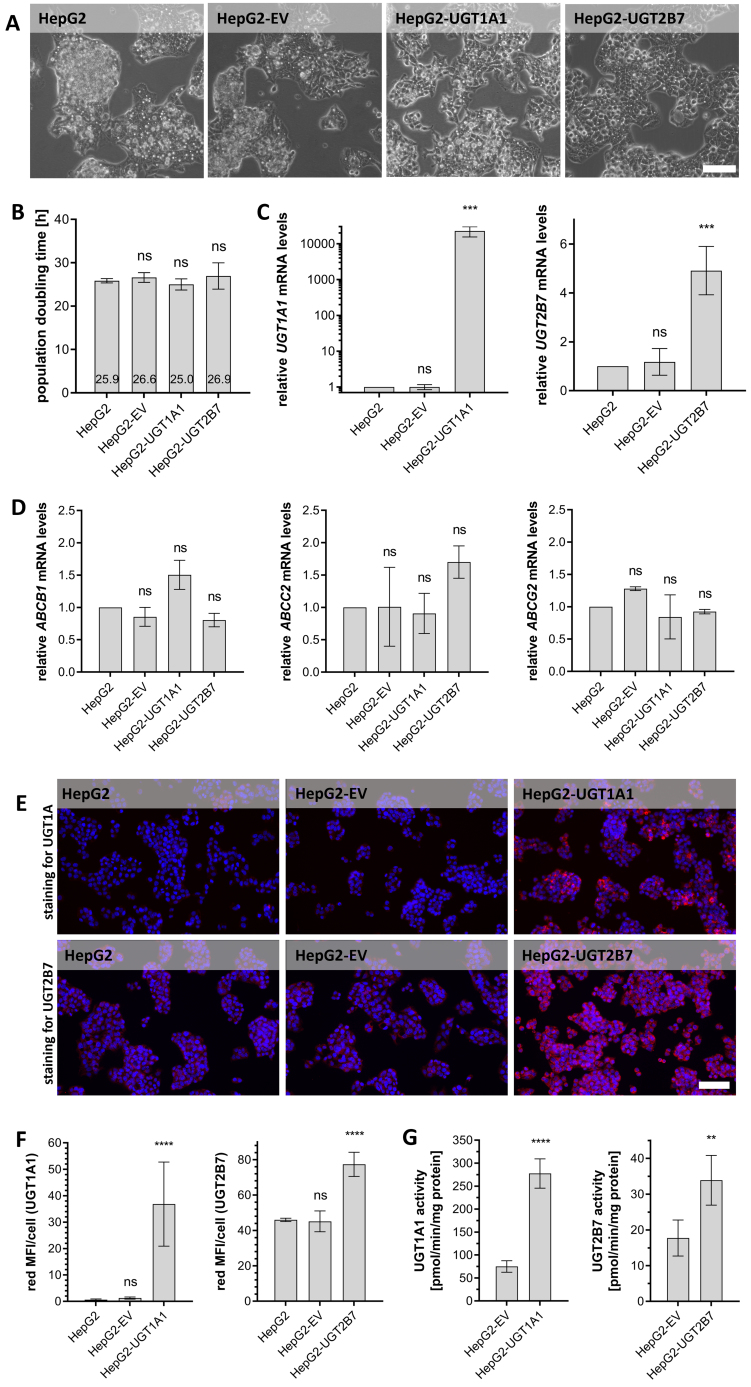
Phenotypic characterisation of HepG2-EV, -UGT1A1 and -UGT2B7 cell clones. (A) Representative phase contrast microscopy images of HepG2 cell clones and HepG2 parental cells cultured as exponentially growing monolayers. Scale bar: 100 µm; (B) PDT of exponentially growing cultures of HepG2-UGT/-EV clones and parental HepG2 cells. No statistically significant differences were identified using one-way ANOVA and Tukey’s multiple comparisons test with three biological replicates; (C) Relative *UGT1A1* (left) and *UGT2B7* (right) mRNA expression levels in HepG2-UGT/-EV cells determined by qRT-PCR. Statistical analysis was conducted using one-way ANOVA followed by Tukey’s test (comparing to parental HepG2 cells) with three biological replicates; (D) Relative *ABCB1*, *ABCC2*, and *ABCG2* mRNA expression levels in HepG2-UGT/-EV cells as determined by qRT-PCR. Statistical analysis was conducted using one-way ANOVA followed by Tukey’s test (compared to parental HepG2 cells, which served as a reference) with three biological replicates; (E) Protein expression of UGT1A1 and UGT2B7 (visualised by red fluorescence) in HepG2-UGT/-EV clones and parental HepG2 cells as determined by immunofluorescence staining with DAPI counterstaining to visualize cell nuclei (blue fluorescence); representative images are shown. Scale bar: 100 µm; (F) Quantification of UGT1A1 (left) and UGT2B7 (right) immunofluorescence in HepG2-UGT/-EV cells, expressed as mean fluorescence intensity (MFI; red channel) per cell; (G) Specific UGT activity in microsomal fractions prepared from HepG2-EV, -UGT1A1 and -UGT2B7 cell clones as determined by the fluorometric UGT Activity Assay/Ligand Screening Kit. Statistically significant differences were identified using a two-tailed unpaired *t*-test with four independent replicates. Significance levels are indicated as follows: ns = not significant (*P* > 0.05), ^**^P < 0.01, ^***^P < 0.001 and ^****^P < 0.0001. PDT: Population doubling times; ANOVA: analysis of variance; mRNA: messenger RNA; qRT-PCR: quantitative real-time polymerase chain reaction; DAPI: 4′,6-diamidino-2-phenylindole; MFI: mean red fluorescence intensity; UGT: uridine diphosphate glucuronosyltransferase.

UGT-overexpressing cell lines and EV control cells were generated from HepG2 cells because their generally low endogenous expression of UGT1A1 and UGT2B7^[14]^ makes it easy to detect ectopic overexpression. The newly generated HepG2-UGT1A1 and UGT2B7 cells were characterised by determining their PDT, by qRT-PCR, and by immunofluorescence staining to assess *UGT*/UGT mRNA and protein expression. As shown in Figure 1A, the new UGT-overexpressing cell lines did not differ morphologically from the parental HepG2 cells. PDT ranged from 25.0 (HepG2-UGT1A1) to 26.9 h (HepG2-UGT2B7), with no statistically significant differences between cell sublines [Figure 1B]. The parental HepG2 and HepG2-EV cells had comparable levels of *UGT1A1* and *UGT2B7* mRNA. However, *UGT1A1* mRNA levels in HepG2-UGT1A1 cells were significantly (2.3 × 10^4^-fold) higher than in the parental cells [Figure 1C, left]. The overexpression of UGT2B7 in HepG2-UGT2B7 cells was less pronounced (4.9-fold higher than in the parental cells) but still significant [Figure 1C, right]. We also used qRT-PCR to determine whether the cloning procedure affected the expression of the pharmacokinetics/multidrug resistance (MDR)-associated transporters *ABCB1*, *ABCC2*, and *ABCG2*, verifying that their expression was unchanged in all the new cell lines [Figure 1D]. The protein expression levels of UGT1A1 and UGT2B7 followed the trend seen for mRNA [Figure 1E and F]. The parental and EV cells exhibited negligible UGT1A1 expression, with MFI/cell values of just 0.6 ± 0.3 and 1.3 ± 0.4, respectively, but the HepG2-UGT1A1 cells produced significantly stronger UGT1A1 immunofluorescence signals, with an MFI/cell value of 36.8 ± 15.4 [Figure 1F, left]. Both the parental and HepG2-EV cells produced detectable UGT2B7 fluorescence signals, with an MFI/cell of 46.0 ± 0.8 and 45.1 ± 5.3, respectively, but HepG2-UGT2B7 cells produced a significantly stronger signal, with an MFI/cell of 77.4 ± 6.3 [Figure 1F, right]. To functionally confirm the overexpression of both UGT enzymes, their glucuronidation activity in the new HepG2 models was assessed using a fluorometric UGT activity assay [Figure 1G]. In accordance with the qRT-PCR and immunofluorescence data, both transduced cell models displayed significantly higher enzymatic activity than the corresponding EV controls. HepG2-UGT1A1 cells exhibited the strongest increase, with 3.7-fold higher specific UGT activity than the EV control [Figure 1G, left]; HepG2-UGT2B7 cells exhibited a 1.9-fold increase over the EV control [Figure 1G, right]. In addition, the basal activity of UGT1A1 in EV cells was 4.2-fold higher than that of UGT2B7 [Figure 1G]. These findings confirm the successful overexpression and functional activity of UGT1A1 and UGT2B7 in the new HepG2 cell lines.

### UGT1A1 and UGT2B7 overexpression weakens the cytostatic effects of epirubicin, etoposide, and SN-38

**Figure 2:**
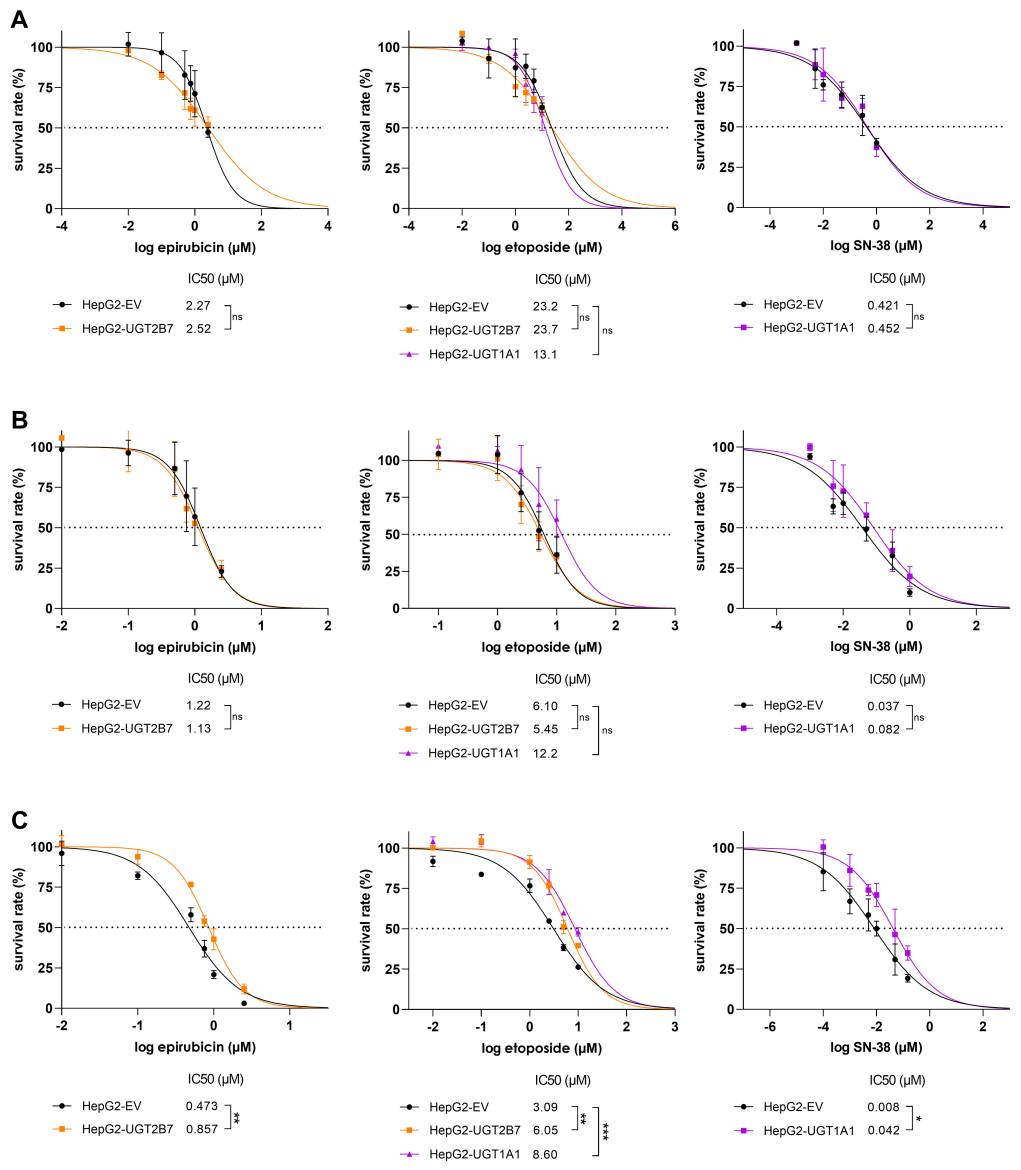
Survival rates of HepG2-EV, HepG2-UGT2B7, and HepG2-UGT1A1 cells after treatment with epirubicin, etoposide, or SN-38. Cells were treated for (A) 24 h, (B) 48 h, or (C) 72 h. Cell viability was assessed using the CellTiter-Glo® 2.0 Cell Viability Assay. IC_50_ values were calculated and then statistically compared using a two-tailed unpaired *t*-test or one-way ANOVA followed by Tukey’s test for multiple comparisons with three biological replicates. Significance levels are denoted as follows: ns = not significant (*P* > 0.05), ^*^P < 0.05, ^**^P < 0.01, and ^***^P < 0.001. ANOVA: analysis of variance.

We initially investigated whether overexpressing UGT isozymes that were previously identified as potential resistance drivers would influence cellular sensitivity towards epirubicin, etoposide, and SN-38. As shown in Figure 2, the IC_50_ values of these drugs in HepG2-EV cells did not differ significantly from those in the UGT-overexpressing lines after 24 or 48 h treatments [Figure 2A and B]. However, after 72 h of treatment the IC_50_ values in HepG2-UGT1A1 and HepG2-UGT2B7 cells were significantly higher than in HepG2-EV [Figure 2C]. The smallest IC_50_ fold change at this stage was 1.81, which was observed for epirubicin in HepG2-UGT2B7 cells. Etoposide exhibited moderate fold changes (1.96 and 2.78 for UGT2B7 and UGT1A1, respectively), while the highest fold change (5.25) was seen for SN-38 in Hep2-UGT1A1 cells. UGT1A1 and UGT2B7 overexpression thus reduced the pharmacological activity of epirubicin, etoposide and SN-38 in a time-dependent manner.

### Specific UGT inhibitors annul the resistance-victim patterns of the studied chemotherapeutics

**Figure 3:**
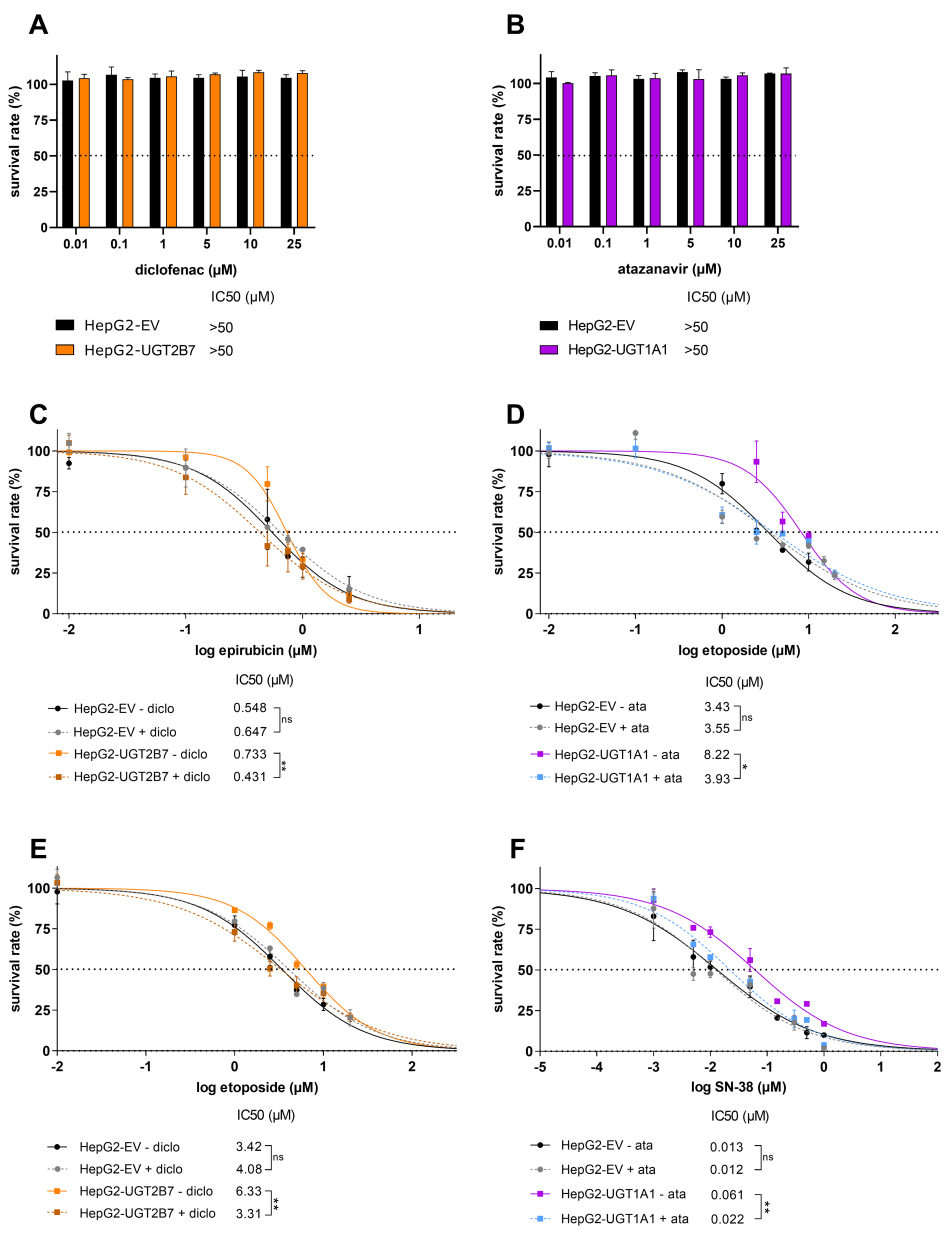
Cell survival rates after treatment with (A) diclofenac and (B) atazanavir; Comparison of cell viabilities after 72 h incubations with epirubicin, etoposide, and SN-38 in HepG2-EV and HepG2-UGT2B7 (C and E) or HepG2-UGT1A1 (D and F) cells with or without the UGT inhibitors diclofenac (diclo; C and E) or atazanavir (ata; D and F) at concentrations of 25 µM. Cell viability was assessed using the CellTiter-Glo® 2.0 Cell Viability Assay. Calculated IC_50_ values were statistically compared using a two-tailed unpaired *t*-test with three biological replicates. Significance is denoted as follows: ns = not significant (*P* > 0.05), ^*^P < 0.05, and ^**^P < 0.01. UGT: Uridine diphosphate glucuronosyltransferase.

The reduced sensitivity of UGT-overexpressing cells to the studied drugs could potentially result from nonspecific factors such as random transgene insertion into a tumour suppressor gene. To exclude such interfering effects, the resistance behaviour was verified in experiments using the potent specific UGT inhibitors diclofenac and atazanavir, which target UGT2B7 and UGT1A1, respectively^[15-17]^. Optimal concentrations of the inhibitors were determined by performing a viability test [Figure 3A and B]. Neither diclofenac nor atazanavir had any toxic effect on HepG2 cells at concentrations up to 25 µM. This concentration, which is known to potently inhibit the targeted UGT isoforms^[15-17]^, was therefore used in subsequent experiments.

To confirm that the tested cytostatics are resistance victims, HepG2 cells were treated with epirubicin, etoposide, or SN-38 with and without UGT inhibitors for 72 h. Adding the UGT2B7 inhibitor diclofenac to epirubicin in HepG2-UGT2B7 cells caused a statistically significant 1.70-fold reduction in the IC_50_ of epirubicin, suggesting that epirubicin is a resistance victim with limited sensitivity [Figure 3C]. More pronounced fold changes were observed upon combined treatment of HepG2-UGT1A1/-UGT2B7 cells with atazanavir/diclofenac and etoposide, which reduced the IC_50_ of etoposide by around 50% and thus confirmed the role of UGTs in resistance to this cytotoxic drug. Importantly, these inhibitors did not significantly affect the IC_50_ in HepG2-EV cells [Figure 3D and E]. Combined treatment with SN-38 and atazanavir in HepG2-UGT1A1 and HepG2-EV clones caused an even stronger (almost three-fold) reduction in IC_50_ in the UGT1A1-expressing cells [Figure 3F].

### UGT1A1-mediated SN-38 resistance can be reversed by TKIs using a dual-activity modulation approach

Dual-activity modulation is a promising strategy for combating cytostatic resistance mediated by ATP-binding cassette (ABC) drug efflux transporters. It is based on the idea that certain novel targeted anticancer drugs can eliminate resistant malignancies via two mechanisms: they exert their own specific antineoplastic effects, but also increase the accumulation of resistance-victim chemotherapeutics by inhibiting ABC transporters. This results in synergistic anticancer activity that occurs selectively in cancer cells due to the targeted nature of the dualactivity modulator^[18]^. We sought to apply this principle to UGT-mediated resistance and explore the possibility that new targeted anticancer drugs designed to inhibit UGTs could have synergistic activity based on the combination of their own anticancer effects and the simultaneous inhibition of etoposide or SN-38 metabolism. Given the lack of recognized potent UGT2B7 inhibitors, we focused solely on UGT1A1 in these studies.

**Figure 4:**
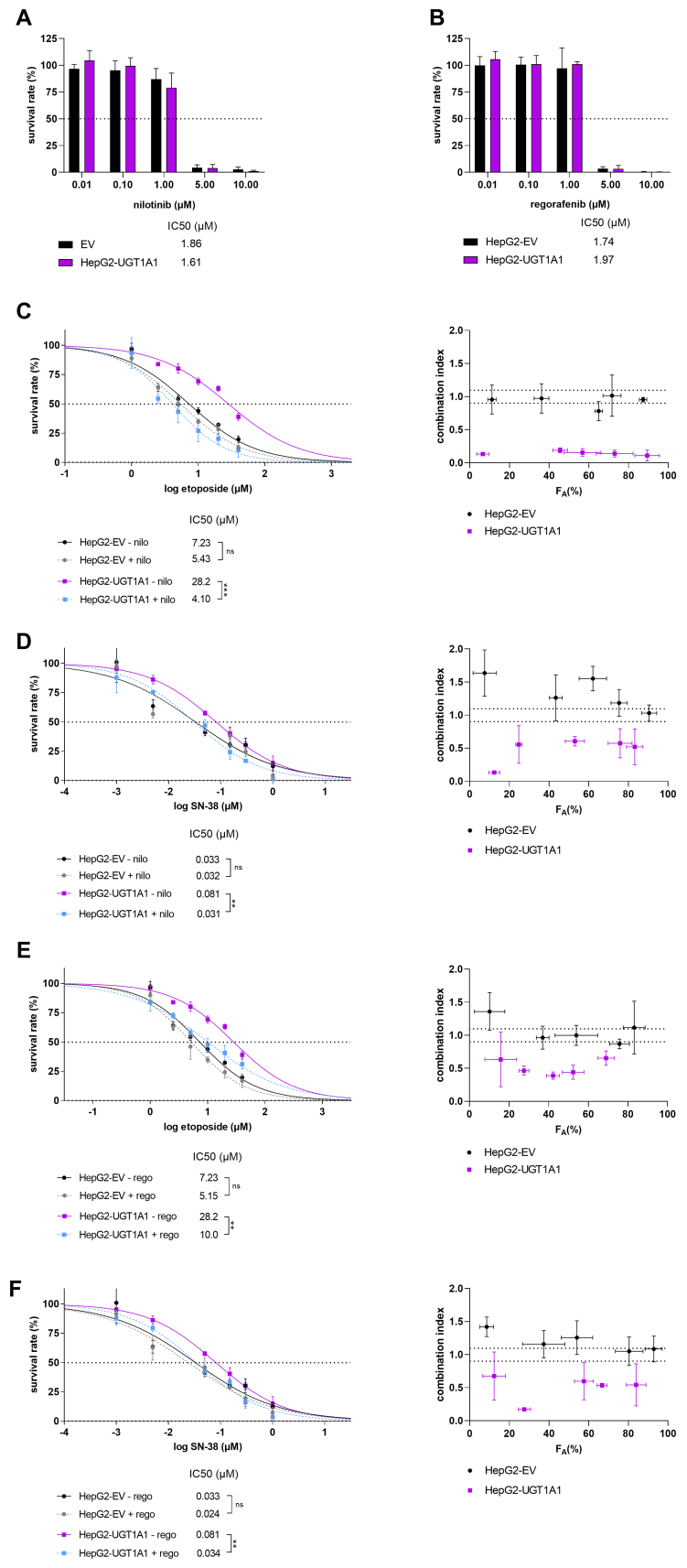
Survival rates for HepG2-EV and HepG2-UGT1A1 cells after treatment with (A) nilotinib and (B) regorafenib; Cell viabilities after 72 h treatment with etoposide (C and E) or SN-38 (D and F) with and without the addition of 1 µM nilotinib (nilo; C and D) or regorafenib (rego; E and F) to inhibit UGT activity. Cell viability was assessed using the CellTiter-Glo® 2.0 Cell Viability Assay. Cell viability curves were constructed from normalized raw data, and the resulting IC_50_ values were statistically compared using a two-tailed unpaired *t*-test with three biological replicates (left). Significance levels are denoted as follows: ns = not significant (*P* > 0.05), ^**^P < 0.01, and ^***^P < 0.001. The outcomes were transformed into F_A_ values (representing the percentage of cells killed by the tested drug concentrations) for use in a Chou-Talalay analysis (right), which classifies drug combination treatments as synergistic (CI < 0.9), additive (CI = 0.9-1.1), or antagonistic (CI > 1.1). UGT: Uridine diphosphate glucuronosyltransferase; CI: combination index.

Two potential dual-activity modulators were investigated in these resistance-reversal experiments: the TKIs nilotinib and regorafenib. Both drugs are potent UGT1A1 inhibitors - nilotinib exhibited a K_i_ of 0.286 μM in human liver microsomes and 0.079 μM against recombinant UGT1A1^[19]^, while regorafenib also strongly inhibited UGT1A1, with submicromolar K_i_ values of 0.020 to 0.480 µM^[20]^. Figure 4 shows that both nilotinib and regorafenib significantly sensitized HepG2-UGT1A1 cells to etoposide and SN-38 at non-toxic clinically relevant concentrations. Specifically, nilotinib caused 6.88- and 2.61-fold reductions in the IC_50_ values of etoposide and SN-38, respectively [Figure 4C and D, left]. The corresponding IC_50_ reductions observed with regorafenib were 2.82- and 2.38-fold, respectively [Figure 4E and F, left]. Importantly, co-treatment with nilotinib or regorafenib did not affect the sensitivity of HepG2-EV cells to etoposide or SN-38, strongly supporting the key role of UGT1A1 inhibition in the observed chemo-sensitisation. To verify this conclusion, the combined treatments were analysed with Chou-Talalay’s CI method, which uses a mathematical algorithm to quantitatively characterise drug combination treatments. Antagonistic or additive effects were observed across most of the F_A_ range in HepG2-EV cells, whereas synergistic effects were observed for the nilotinib and regorafenib combinations in UGT1A1-overexpressing HepG2 cells [Figure 4C-F, right].

### UGT1A1 and transporter expression in primary-like human hepatocytes

**Figure 5:**
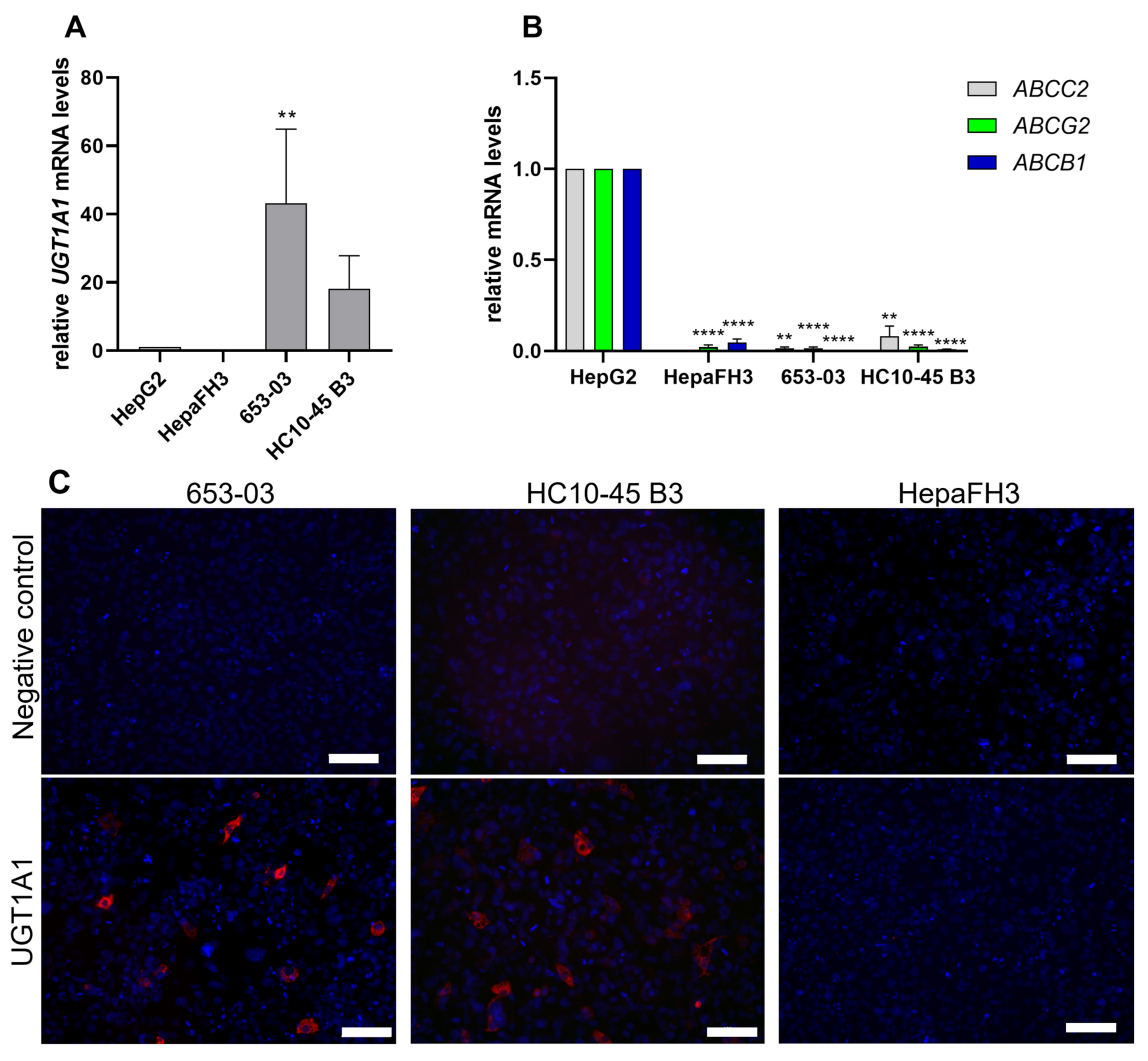
Relative mRNA expression levels of (A) *UGT1A1* and (B) the pharmacokinetics/MDR-associated drug efflux transporters *ABCC2*, *ABCG2*, and *ABCB1* in primary-like upcyte hepatocytes from three donors as determined by qRT-PCR. HepG2 cells served as a reference. The data were statistically analysed by one-way ANOVA followed by Tukey’s post-test for multiple comparisons (using HepG2 as the reference) with three biological replicates. Significance levels are denoted as follows: ^**^P < 0.01 and ^****^P < 0.0001; (C) Protein-level expression of UGT1A1 (visualized by red fluorescence) in 653-03, HC10-45 B3 and HepaFH3 upcyte hepatocytes as determined by immunofluorescence staining. Cell nuclei were counterstained with DAPI (generating blue fluorescence). Scale bar: 100 µm. mRNA: Messenger RNA; MDR: multidrug resistance; qRT-PCR: quantitative real-time polymerase chain reaction; ANOVA: analysis of variance; DAPI: 4′,6-diamidino-2-phenylindole.

We next sought to confirm the role of UGT1A1 as a mediator of drug resistance in a more physiologically relevant complex model. To this end we used primary-like human hepatocytes generated from human biopsies using upcyte technology. This advanced technology creates proliferation-competent primary human hepatocytes while conserving crucial physiological features such as phase I and phase II enzyme expression^[10,11]^. Upcyte hepatocytes from three donors (HepaFH3, 653-03, and HC10-45 B3) were screened to assess their *UGT1A1*/UGT1A1 mRNA and protein levels as well as mRNA expression of efflux transporters with important roles in pharmacokinetics/drug resistance whose activity could influence the results obtained^[21,22]^. Figure 5A shows that *UGT1A1* mRNA levels in HC10-45 B3 and 653-03 cells were 43.2-fold and 18.2-fold higher, respectively, than those in HepG2 cells. Conversely, *UGT1A1* levels in HepaFH3 were near the detection limit. Additionally, the pharmacokinetics/MDR-associated efflux transporters *ABCC2*, *ABCG2*, and *ABCB1* were expressed at significantly lower levels in the 653-03, HC10-45 B3, and HepaFH3 cells than in HepG2 cells, highlighting their negligible influence on drug resistance in the selected models [Figure 5B]. Immunofluorescence staining was performed to verify protein-level UGT1A1 expression in the new upcyte hepatocytes [Figure 5C]. HepaFH3 cells showed no UGT1A1 signal, but 653-03 and HC10-45 B3 cells showed clear UGT1A1 signals, confirming the PCR findings.

### UGT1A1 inhibition potentiates SN-38 activity in a physiologically relevant hepatocyte model

**Figure 6:**
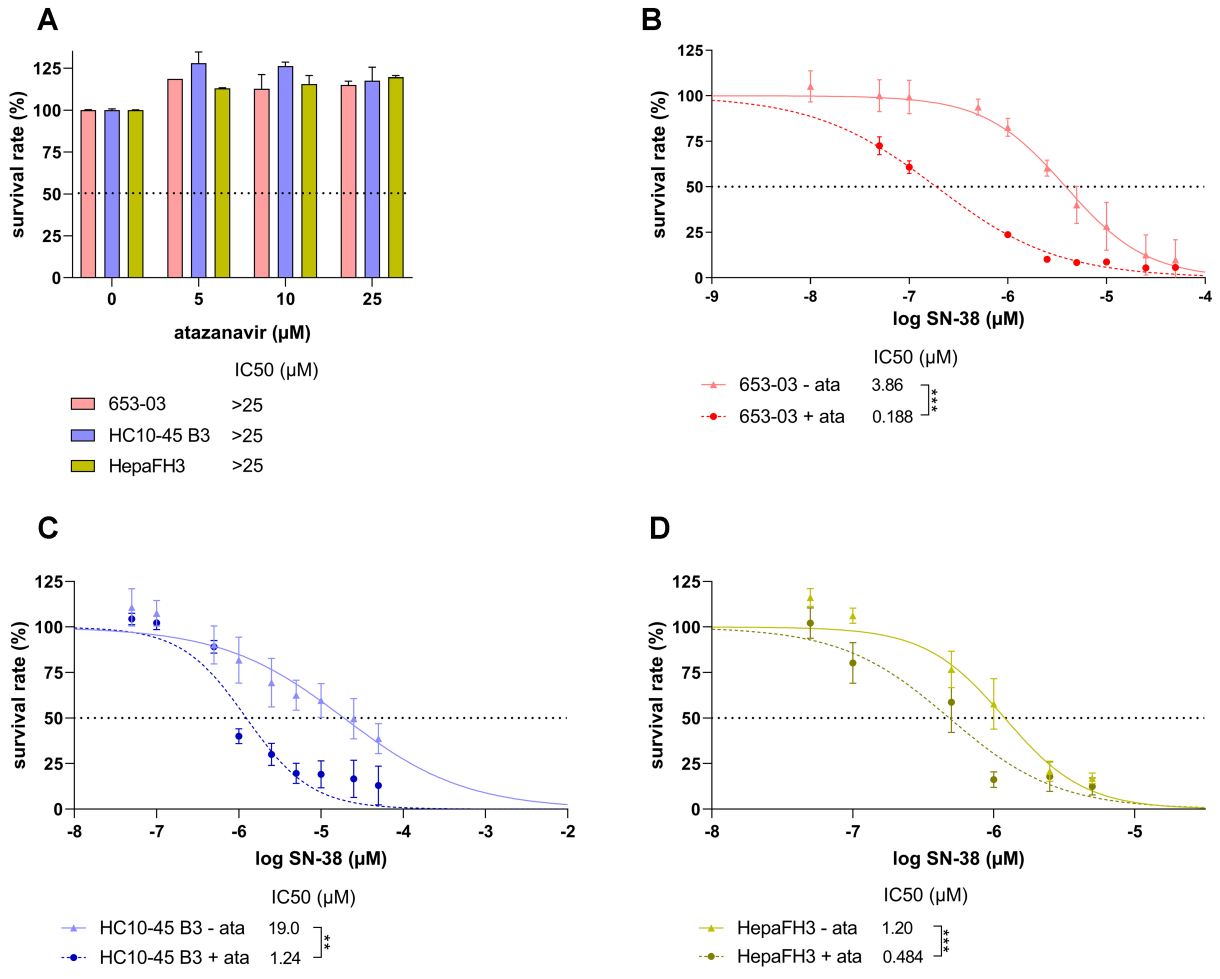
Cell survival rates of primary-like upcyte hepatocytes treated for 72 h with atazanavir (ata) alone (A), and for 653-03 (B), HC10-45 B3 (C), and HepaFH3 (D) cells treated with SN-38 alone and in combination with atazanavir. Cell viability was assessed using the CellTiter-Glo® 2.0 Cell Viability Assay. Calculated IC_50_ were statistically compared using a two-tailed unpaired *t*-test with three biological replicates. Significance levels are denoted as follows: ^**^P < 0.01 and ^***^P < 0.001.

To confirm the status of UGT1A1 as a targetable resistance driver, the atazanavir inhibition assay was repeated using the primary-like upcyte hepatocytes. In preliminary experiments, the cells were treated with atazanavir for 72 h to check for possible toxic effects. No such effects were observed at therapeutically relevant concentrations; the IC_50_ for atazanavir was determined to be > 25 µM [Figure 6A]. Therefore, as in the experiments with transduced HepG2 cells, atazanavir was used at a concentration of 25 µM in the combined treatments with SN-38 [Figure 6B-D].

Adding atazanavir significantly reduced the IC_50_ values for SN-38 in upcyte hepatocytes from all three donors. Specifically, 20.5-, 15.4- and 2.48-fold IC_50_ reductions were observed for 653-03, HC10-45 B3, and HepaFH3 [Figure 6B-D, respectively]. Notably, the IC_50_ shift in primary-like HepaFH3 cells was almost an order of magnitude smaller than those in 653-03 and HC10-45 B3 cells. This aligns well with the finding that HepaFH3 cells have much lower *UGT1A1*/UGT1A1 levels than their 653-03 and HC10-45 B3 counterparts [Figure 5].

## DISCUSSION

Drug resistance is a major obstacle to the successful pharmacotherapy of oncological patients. Its onset is driven by both pharmacodynamic and pharmacokinetic mechanisms. The roles of drug transporters in resistance are well understood, but few studies have provided direct evidence about the roles of drug metabolising enzymes. Here, we investigated UGT enzymes as possible drivers of cytostatic resistance and showed that their influence can be suppressed using a targeted dual-activity modulation approach.

In comparative ATP viability assays using single-gene transduced HepG2 models, UGT1A1 and/or UGT2B7 significantly affected the pharmacodynamic activity of three drugs: epirubicin, etoposide, and SN-38 (the active metabolite of irinotecan). Verification experiments using the UGT inhibitors atazanavir and diclofenac confirmed all three drugs to be resistance victims, with SN-38 exhibiting the most pronounced effect. These resistance patterns only became significant after 72 h of drug exposure, which aligns with the measured PDT of the HepG2 cells and the primarily phase-specific action of the studied topoisomerase inhibitors; direct toxicity resulting from DNA intercalation is believed to be a less important secondary mechanism in their activity^[23]^. Despite being statistically significant, the observed resistance patterns were comparatively weak. This is consistent with our previously reported conclusion that drug-metabolising enzymes are important but generally less potent drivers of pharmacokinetic resistance than ABC drug efflux transporters^[24,25]^. However, our experiments with upcyte hepatocytes showed that UGT-mediated resistance can manifest even in cells with physiological enzyme expression, suggesting a potentially significant impact *in vivo*. To date, most studies on the possible role of UGT1A1 in tumour resistance to SN-38 have been clinical trials involving cancer patients or experimental studies using complex models. These studies have found or predicted that inhibition-based drug-drug interactions (mediated by silybins, gefitinib, and erlotinib) can increase SN-38’s plasma AUC and thus potentiate its anticancer effects^[26-28]^. In addition, several publications have discussed the influence of *UGT1A1* polymorphisms on the metabolism and toxicity/efficacy of SN-38. Notably, some patients, especially from Asia, express UGT1A1*6 or *28 gene variants with impaired activity, which increases the risk of side-effects during SN-38 treatment and thus necessitates dosage adjustments. This and similar observations illustrate the need to genotype patients before applying UGT1A1-dependent therapies^[29-32]^. While some studies have previously examined variations in systemic SN-38 disposition caused by differences in UGT1A1 levels, polymorphisms, and drug–drug interactions (DDIs) in the intestine and liver, this is the first work to examine the interaction between SN-38 and UGT1A1 inhibition at the intratumoural level. Importantly, unlike earlier studies, our initial experiments used “single gene”-transduced models, eliminating interfering effects that may exist when using multienzyme models. Moreover, we documented resistance patterns at clinically relevant SN-38 concentrations within the ranges seen in plasma from cancer patients (for which the C_max_ is 74.7 nM at the average dosage)^[33]^. This suggests that our findings may be translatable to *in vivo* contexts such as colorectal cancer, for which irinotecan is a first-line chemotherapy.

The feasibility of overcoming systemic pharmacokinetic resistance using non-toxic inhibitors was studied intensively during the first decade of the 21st century. These efforts focused mainly on modulating ABC transporters but failed: the developed inhibitors not only increased the accumulation of cytotoxic drugs in tumours but also raised their plasma levels by enhancing absorption and blocking elimination, leading to unacceptable systemic toxicity^[34]^. Similar drawbacks were encountered in efforts to exploit enzyme inhibition: co-administration of the non-toxic CYP34A inhibitor ketoconazole with docetaxel (a substrate victim of CYP3A4-mediated resistance) reduced docetaxel clearance by 49% in cancer patients^[35]^. While this may have increased treatment efficacy, it also greatly raised the risk of febrile neutropenia, negating the benefit. Dual-activity modulation of intratumoural MDR has emerged as a promising alternative to these earlier strategies^[18]^. A key feature of this approach is that the anticancer properties of dual activity modulators can be highly selective, making it possible to induce synergistic responses in tumour cells only. This enables the use of lower drug doses while avoiding the limitations of earlier non-toxic inhibitors. Most work on dual-activity MDR modulation has focused on ABC transporters^[18]^; the results presented herein demonstrate its applicability to UGT-mediated resistance. Our drug combination studies showed that both regorafenib and nilotinib can induce the desired synergistic responses: both agents potentiated etoposide/SN-38 activity in UGT1A1-expressing cells. Importantly, the synergistic action was observed at clinically relevant concentrations (C_max_ of 7.15 and 3.10 µM for regorafenib and nilotinib, respectively)^[36,37]^, suggesting potential translatability to living tissues. Elevated UGT1A1 expression has been observed in liver cancer but also in colorectal, breast, stomach, and biliary cancers^[14,38,39]^. As regorafenib and irinotecan (the SN-38 prodrug) are both frequently used to treat colorectal cancer^[40,41]^, combined treatment with both drugs could have strong beneficial effects. This hypothesis is supported by the results of a clinical trial in colorectal cancer patients reported by Schultheis *et al.*, in which combined treatment with regorafenib and irinotecan was significantly more effective than irinotecan alone, with tolerable side effects^[42]^. Our findings offer a potential molecular explanation for this beneficial outcome. Multiple studies have shown that UGT1A1 inhibition by nilotinib impairs SN-38 glucuronidation in liver microsomes^[19,43]^. However, the impact of this interaction on the cytostatic efficacy of SN-38 has not previously been investigated. It has also been shown that regorafenib and nilotinib can synergistically boost the efficacy of etoposide, which is used in stomach cancer therapy^[41]^. As noted above, stomach cancer exhibits significant UGT1A1 expression^[38]^ and may thus be a good target for dual-activity MDR modulation. We found no studies on combined treatment with etoposide and regorafenib, but the combination of nilotinib and etoposide reportedly exhibited synergistic effects in LAMA84 cells^[44]^ that could plausibly be at least partly driven by UGT1A1 inhibition. Some drug groups other than the TKIs tested in this work could also be effective dual-activity MDR modulators. For example, tucatinib and osimertinib reportedly inhibited UGT1A1-mediated SN-38 glucuronidation^[45,46]^. Similarly, erlotinib, gefitinib, and imatinib were identified as UGT1A1 inhibitors^[47]^. Combined treatment with erlotinib and SN-38 reversed ABCG2-mediated resistance to SN-38^[48,49]^ while gefitinib and imatinib increased the effects of SN-38 in small cell lung cancer^[50]^. In all these studies, the positive results were attributed to the inhibition of ABC transporters and a subsequent increase in SN-38 accumulation. The results presented here offer the first mechanistic evidence that this strategy may also operate in part by suppressing UGT-mediated MDR. There have also been clinical trials on sorafenib, which is used to treat multiple cancer types, including hepatocellular carcinoma. Some of these studies explored its combined use with irinotecan to improve treatment efficacy across different tumour types, revealing the that combined treatment increased efficacy with tolerable side effects^[51,52]^. The authors invoked pharmacodynamic principles to explain this promising outcome, focusing on the capacity of sorafenib to block Raf signalling while irinotecan inhibits topoisomerase I. However, sorafenib is also a potent UGT1A1 inhibitor^[53,54]^. Given our results, we therefore hypothesise that sorafenib-mediated inhibition of irinotecan metabolism may be at least partly responsible for the positive outcomes recorded in these trials. Sorafenib is thus another potential dual activity chemosensitiser. These results indicate that successful implementation of dual activity modulation will require both genotyping of patients for UGT1A1 expression and careful consideration of the primary targets of any TKIs that are to be used. Targeted distribution technologies could further enhance the clinical applicability of this approach.

In the final part of our study, we verified the results obtained using “single gene”-transduced cells in a more physiologically relevant model. To this end, we performed experiments using primary-like upcyte hepatocytes originating from healthy liver tissue that can proliferate *in vitro* like cancer cells despite being non-tumourigenic. Unlike other liver models such as HepG2 cells, these primary human donor-derived cells retain physiologically relevant enzymatic machinery^[55]^. These experiments confirmed that UGT1A1 can contribute significantly to MDR even in a competitive multienzyme environment and is targetable with UGT inhibitors. This finding is important because drugs are often metabolized by multiple enzymes, so the inhibition of one enzyme involved in MDR is often compensated by changes in the activity of others. Similarly, chemotherapeutics often display overlapping substrate affinity with multiple ABC transporters, so blocking the function of one will not always have the desired effect^[34,56,57]^. Despite this, the upcyte findings reinforced the designation of UGT1A1 as target for improving chemotherapy outcomes in oncological patients.

Our work has a few limitations. As noted previously, the observed resistance patterns were significant but their magnitude was comparatively modest, especially for epirubicin and etoposide. Animal and human studies, which this work lacked, will be needed to verify the role of UGT enzymes in chemotherapeutic resistance *in vivo*. In addition, colorectal cancer models would be more suitable than hepatic ones given irinotecan’s primary indication. Our initial “blind” choice of HepG2 cells as a model was guided by established practice: these cells are a recognized pre-model for metabolismoriented studies. We overcame their limited drug metabolizing activity by overexpressing the UGTs under investigation and confirmed our initial findings by performing experiments with more primary-like and physiological upcyte cells. However, future translational validation studies should include colorectal models to better mimic clinical reality.

In conclusion, we have shown that high UGT1A1 expression confers resistance to epirubicin, etoposide, and SN-38. In addition, our data reveal that this pharmacokinetic MDR mechanism can be reversed using an innovative dual-activity modulation approach. With *in vivo* confirmation, this strategy could be translated to create effective and safe combination regimens for treating oncological patients.

## DECLARATIONS

### Availability of data and materials

All data can be found in the article. The raw data supporting the findings of this study are available from the corresponding author upon reasonable request.

### AI and AI-assisted tools statement

During the preparation of this manuscript, the AI tool Claude 3.7 (Sonnet) (via Perplexity AI, web interface) was used solely for language editing. The tool did not influence the study design, data collection, analysis, interpretation, or the scientific content of the work. All authors take full responsibility for the accuracy, integrity, and final content of the manuscript.

### Copyright

© The Author(s) 2026.
